# Significance of Tumor Microenvironment for Regulating Pancreatic Cancer

**DOI:** 10.3390/cancers15092482

**Published:** 2023-04-26

**Authors:** Hideaki Ijichi

**Affiliations:** 1 Clinical Nutrition Center, Graduate School of Medicine, The University of Tokyo, 7-3-1 Hongo, Bunkyo-ku, Tokyo 113-8655, Japan; hideij@g.ecc.u-tokyo.ac.jp; Tel.: +81-3-3815-5411; 2 Department of Gastroenterology, Graduate School of Medicine, The University of Tokyo, 7-3-1 Hongo, Bunkyo-ku, Tokyo 113-8655, Japan

Pancreatic cancer is the most lethal common cancer in the world. Although the accumulation of intensive research and clinical efforts in the last three decades has definitely advanced the knowledge of and therapies for this disease, the overall 5-year survival rate still remains around 10%. The incidence and deaths have been progressively increasing; as a result, pancreatic cancer is the third leading cause of cancer deaths in the United States, and it is thought that it will be the second by 2040 [1,2,3]. Of note, the figures related to incidence and deaths are very close, which also indicates the high lethality of this disease. Thus, it is not too much to say that improvement of the prognosis of pancreatic cancer, especially of the most common type, pancreatic ductal adenocarcinoma (PDAC), is one of the most important unmet medical needs.

PDAC exhibits a very characteristic tumor histology: the tumor tissue contains relatively few cancer cells, but abundant stromal components, especially fibrosis including fibroblasts and the extracellular matrix called “desmoplasia”, is the representative feature. In addition, it contains immune-inflammatory cells, blood and lymphatic vessels, neural cells, etc., all of which form the PDAC tumor microenvironment (TME) [4]. Therefore, PDAC is basically composed of heterogeneous cellular and non-cellular populations, which suggests that the pathobiology and malignant potential of PDAC are highly influenced by this characteristic TME. It is also well known that PDAC is a hypovascular tumor. Taken together with dense fibrosis, it is easy to see that the TME of PDAC acts as the high defensive wall, disturbing the drug delivery and protecting the PDAC cells from the anti-cancer therapies. Recently, cancer immunotherapy has been introduced into the clinical practice of many cancer types and has changed the therapeutic strategy and prognosis. However, PDAC is also refractory to the immune checkpoint inhibitors, which has been attributed to the low tumor mutation burden with relatively little cytotoxic effector T cell infiltration and an immunosuppressive environment, which is known as a “cold” tumor [5]. 

The advance of next-generation sequencing (NGS) technology has developed cancer genomics and precision medicine. Currently, oncogene panel screening can detect actionable mutation; thereby, specific molecular targeting therapy can be applied to the patients [6]. PDAC is considered to develop via multi-step carcinogenesis along with the accumulation of genetic alterations of *KRAS*, *CDKN2A*, *TP53*, *SMAD4*, and others [7]. Whole-exome sequencing as well as NGS revealed that the four genes were the most frequently mutated in PDAC [8,9], and the four were named the “Big 4” genes. Since the driver effect on PDAC carcinogenesis of these genes was confirmed by genetically engineered mouse studies [10,11,12,13,14], the “Big 4” genes can be good therapeutic targets for PDAC; however, KRAS has been an undruggable target for a long time, and no specific therapies targeting the other three genes have been developed yet. 

In addition, cancer genomics and precision medicine has improved the prognosis of many cancer types, including PDAC [15], although it was reported that the actionable mutations were found in 28% of PDAC patients screened, and only 6.8% of PDAC patients underwent the specific molecular targeted therapy [15], which suggests that the benefit of current precision medicine in PDAC is limited. To improve the prognosis of PDAC patients, it might be important to explore not only the cancer genome, but also the characteristic TME. 

KRAS has been an undruggable target for a long time [16]; however, it is now changing. Recently, the KRAS G12C-specific inhibitor has already come into clinical practice as the first mutant allele-specific KRAS inhibitor [17]. However, unfortunately, G12C mutation is rare in PDAC, and the benefits are also limited [18]. The most frequent pattern of *KRAS* mutation in PDAC is KRAS G12D (41%), and G12V is the second (34%) [16]; hence, the specific inhibitors of them have been long awaited. Very recently, the KRAS G12D-specific inhibitor was developed and reported to have a potent therapeutic effect on PDAC [19,20]. Currently, the KRAS G12D inhibitor is under a phase 1/2 clinical trial, and a promising clinical impact is anticipated in the near future. Therefore, the situation is definitely improving compared to before; however, considering the acquired resistance observed in the treatment with the KRAS G12C inhibitor [21], the combination of targeting KRAS and regulating TME might be a more promising strategy for conquering PDAC. It is reported that a more aggressive subtype of PDAC is enriched with gene sets of the activated KRAS signaling pathway; moreover, the TME is also reported to be involved in the PDAC subtype formation [22].

In this Special Issue entitled “Tumor Microenvironment and Pancreatic Cancer”, various aspects of PDAC TME are discussed. As described above, the TME of PDAC is composed of heterogeneous cellular and non-cellular populations. In addition, it is known that there is also heterogeneity in the same cell type, for example, M1 and M2 types in macrophages, which might be associated with functional heterogeneity: tumor-suppressive or tumor-supporting. The dense stroma and fibrosis have been believed to support PDAC cells in the TME; however, it is not so simple. Surprisingly, studies depleting fibroblasts or decreasing stromal volume resulted in more aggressive PDAC phenotypes and worsened the survival of genetically engineered PDAC mice [23,24], which is reminiscent of the functional heterogeneity in cancer-associated fibroblasts (CAFs) as well as the dense stroma, which might have tumor-suppressive roles, not only tumor-supporting roles. Recent single-cell analyses as well as multi-omics analyses revealed that there exist different clusters of CAFs, macrophages, and other immune cells in the PDAC TME [25,26,27]. The various clusters of various cell types are interacting with other clusters or other cell types, including PDAC cells; thereby, the function might be changed temporally and spatially even in the same cell. As the PDAC TME is studied deeper, the heterogeneity seems to become more complicated; however, elucidating and accumulating what is going on in the complicated PDAC TME might be essential to understand and regulate the difficult disease that is PDAC. 

In this Special Issue, CAFs and the extracellular matrix (ECM), impressive features of the PDAC TME, were the most frequently reviewed and discussed [28,29,30,31,32,33,34]. Functional heterogeneity of CAF subtypes was repeatedly described: some might be tumor-promoting, while others might be tumor-suppressive. Masugi described multilayered levels of stromal heterogeneity, which included various CAF subtypes and ECM components, cell-to-cell interactions and niche formations, locoregional and organ-level heterogeneity, and intertumor heterogeneity, and discussed potential opportunities for targeting the stroma [28]. Ando et al. highlighted cancer-restraining CAFs compared with cancer-promoting CAFs and the plasticity between the two, further demonstrating the possibility of therapeutic intervention converting “bad” stroma to “good” stroma [29]. Shinkawa et al. reviewed the tumor-promoting and immunosuppressive CAF subtypes as well as tumor-suppressive and tumor differentiation-related CAF subtypes, and discussed the relationship between CAF subtypes and the immune microenvironment as a critical therapeutic target [30]. Skorupan et al. overviewed the recent and current clinical trials of immune-modulating agents and stroma-targeting agents and described the potent combination of targeting PDAC cells with TME modulation [31]. Wang et al. overviewed ECM physiology in a healthy status as well as in PDAC, and discussed the crucial roles of ECM, focusing on its influence on the cancer stem cell property [32]. Heger et al. analyzed the PDAC stroma after neoadjuvant chemotherapy (NAC) and documented a better prognosis of the patients with low α-smooth muscle actin density, suggesting a difference from the patients who underwent upfront surgery [33]. Ijichi also discussed the CAF heterogeneity along with the prognostic significance of the sub-tumor microenvironment (subTME), a combination of CAF subtypes and various immune-inflammatory infiltrates as well as ECM [34]. 

Rubin et al. discussed the duality and complexity of immune cell functions in the PDAC TME, which might be more complicated than the classical “hot” and “cold” tumor, and the roles of immune microenvironment in the treatment of PDAC [35]. Gorchs et al. demonstrated that regional T cell infiltration into the PDAC tissue was mediated by chemokine signals, and the infiltration might be inhibited via downregulating chemokine receptors on T cells by CAFs [36]. 

Miyabayashi et al. reviewed the current knowledge of the association of PDAC TME and the microbiome [37]. As expected in other cancers, the microbiome is considered to be significantly involved in the pathogenesis of PDAC, and hence might be a potent therapeutic target.

Yamamoto et al. overviewed the unique metabolic property of PDAC, which enables the PDAC cells to thrive in the hypoxic and nutrient-deprived TME. They especially focused on the roles of autophagy, which is implicated in therapeutic resistance, immune evasion, and other various refractory aspects of refractory PDAC, and also noted the metabolic crosstalk between PDAC and stromal cells [38]. 

Takahashi et al. described the significant roles of neural signaling in PDAC. Sympathetic, parasympathetic, and sensory neurons are found in the PDAC TME, and the neural signaling targets not only neural cells, but also tumor cells and immune cells via neural receptors expressed on those cells, thereby regulating tumor growth, inflammation, and anti-tumor immunity, which suggests that the neural signal network can also be the therapeutic target [39].

Lai et al. summarized implications of anesthesia in the PDAC TME, including studies of clinical and non-clinical conditions, and suggested that some anesthetic or analgesic agents might promote PDAC progression [40], which might be associated with the roles of the neural signal network as described by Takahashi et al. [39]. Thus, anesthesiologists might need to reestablish the perioperative anesthetic management of PDAC patients to avoid the tumor-promoting effect.

Various aspects of PDAC TME were discussed in this Special Issue, which definitely help in our understanding of PDAC; moreover, we need to elucidate the underlying mechanisms in PDAC and the TME for the improvement of the patients’ prognosis and to conquer the most lethal cancer in the world.
